# Antibiofilm efficacy of emodin alone or combined with ampicillin against methicillin-resistant *Staphylococcus aureus*

**DOI:** 10.1038/s41598-025-06800-5

**Published:** 2025-07-01

**Authors:** Maoying Zhao, Fuhong Chen, Wei Yang, Tao Yan, Qi Chen

**Affiliations:** Department of Clinical Laboratory, Hangzhou Traditional Chinese Medicine Hospital Affiliated to Zhejiang Chinese Medical University, Hangzhou, 310000 China

**Keywords:** MRSA, Emodin, Ampicillin, Antibiofilm activity, Biotechnology, Microbiology, Infectious diseases

## Abstract

Methicillin-resistant *Staphylococcus aureus* (MRSA) is recognized as a significant global health concern. The development of resistance to a broad spectrum of antibiotics, particularly following biofilm formation, renders conventional therapeutic options for MRSA ineffective. Three MRSA clinical isolates were examined in vitro to assess their biofilm-forming capacity and the disruptive effects on pre-established biofilm (via crystal violet staining and scanning electron microscopy), and quantify extracellular DNA (eDNA) release after exposed to emodin alone or in combination with ampicillin. In addition, real-time PCR was employed to investigate the impact of emodin on the expression of biofilm-related genes in MRSA biofilms. The inhibitory effect of emodin on biofilm formation and disruption was observed in a dose dependent manner. The antagonistic activity of emodin in combination with ampicillin against MRSA biofilms was confirmed through adhesion assays. Real-time PCR analysis revealed that emodin, either alone or in combination with ampicillin, effectively downregulated the transcriptional levels of the biofilm-related genes *fnbpB*, *clfA* and *atlA*, but not *icaA*. In addition, drug treatment resulted in a significant reduction in eDNA release and protein contain in EPS (extracellular polymeric substances), which corresponded to the markedly decreased transcript level of *atlA* and *fnbpB*, respectively. These observations suggest that emodin, either alone or in combination with ampicillin, holds potential as a therapeutic approach for MRSA biofilm-related infections.

**Supplementary Information:**

The online version contains supplementary material available at 10.1038/s41598-025-06800-5.

## Introduction

*Staphylococcus aureus* is one of the most important pathogens responsible for a wide spectrum of diseases, the onset of which can be attributed to its production of various virulence factors^1^. The formation of biofilm by *S. aureus* is an additional pathogenic factor, as biofilms serve as reservoirs for persistent infections^2^including implant-associated infections, chronic wounds, fibrotic lung infections and endocarditis^3^. The special three-dimensional structure of biofilms makes them difficult to be eliminated by antibiotics and host defense molecules, leading to a substantial increase in minimum inhibitory concentrations (MICs) compared to planktonic bacteria^4^. Moreover, the matrix of biofilm provides an ideal environment for closer cell-to-cell contact, which facilitates horizontal gene transfer, including plasmids containing genes resistance to several antibiotics^5^. The widespread use of indwelling medical devices (catheters, joint prostheses, heart stents, etc.) in clinical treatment has contributed to the increased incidences of *S. aureus* biofilm-related infections^6^. Currently, the only approach employed to address such cases is the replacement of colonized devices followed by treatment with antibiotics, which significantly escalates medical costs.

Methicillin-resistant *S. aureus* (MRSA), a multidrug-resistant strain, has emerged as a leading cause of community- and hospital-acquired infections around the world^7^. The ability of MRSA to form biofilms is a crucial factor that can undermine the effectiveness of chemotherapy due to its inherent resistance and the protective polysaccharide barrier it possesses. Unfortunately, MRSA strains exhibit a higher propensity for biofilm formation than methicillin-sensitive *Staphylococcus aureus* (MSSA) strains under conditions of glucose deficiency^8^. Bacteria experience continuous exposure to sub-MICs of antibiotics at the beginning and end of treatment or during prolonged low-dose therapy. In addition, sub-MICs of β-lactam antibiotics induce the release of extracellular DNA (eDNA), an essential biofilm component, and promote biofilm formation in MRSA strains while having no effect on MSSA strains^9^.

Vancomycin is the initial treatment option for MRSA-related infections. In recent years, the susceptibility of vancomycin to MRSA has been reported to dimmish and even disappear, especially with the appearance of vancomycin-intermediate and -resistant *S. aureus* (VISA and VRSA, respectively)^10^. Regarding biofilm-associated infections, several studies have reinforced the evidence of poor efficacy of vancomycin against *S. aureus* biofilms^11,12^. Due to the low efficiency and potential resistance development of resistance against *S. aureus*, efforts have been made to explore suitable combined effects of vancomycin with other antibiotics. However, there is limited evidence supporting the efficacy of the adjunctive use of rifampin and vancomycin, as there was no promise for the treatment of MRSA biofilm-related infections^13^. Similarly, there is an ongoing debate regarding the combined use of vancomycin with other antibiotics, such as linezolid, tigecycline and oxacillin^3^. Daptomycin, a cyclic lipopeptide molecule, has been reported to be effective against VRSA biofilm-associated infections^7^ and capable of eradicating *S. aureus* from an existing biofilm alone^14^ or when used in combination with other drugs^15–17^. However, there have also been reports of the failure of daptomycin against biofilm-producing *S. aureus* strains in vitro and in animal models^18–20^.

Recently, natural antimicrobials, primary derived from plants, have been identified to exhibit antibiofilm properties against *S. aureus*. Nevertheless, the efficacy of these antimicrobials is generally weaker than that of conventional drugs produced by bacteria and fungi. However, natural antimicrobials, unlike conventional antibiotics, do not exert selection pressure on bacteria and can synergistically enhance the activity of other antibiotics. For instance, baicalein or hamamelitannin in combination with vancomycin^21,22^quinine with ciprofloxacin^23^ and xanthohumol with oxacillin^24^ have shown potent synergistic effects. Emodin, a compound extracted from *Polygonum cuspidatum* and *Rheum palmatum*, has been investigated for its ability to inhibit cell proliferation and ER stress^25^prevent obesity and cancer^26^and attenuate Alzheimer’s disease^27^. Additionally, emodin has exhibited significant activity against *Haemophilus parasuis*, *Pseudomonas aeruginosa*, *Streptococcus suis* and even *S. aureus*^28,29^. One study demonstrated that emodin reduced *S. aureus* biofilms by preventing eDNA release and downregulating the expression of biofilm-related genes^30^. However, the synergistic antibiofilm effectiveness of emodin and other traditional antibiotics on MRSA has not been reported.

Therefore, the objective of this study was to evaluate the congruous efficacy of emodin in combination with ampicillin, a wide used β-lactam antibiotics, against biofilm cultures of MRSA and to unveil the mechanism of action.

## Results

### Determination of minimum inhibitory concentrations and minimal bactericidal concentrations in suspension and biofilm

The MICs of emodin alone against these three strains in suspension cells were 16, 32 µg/ml and 32 µg/ml, respectively. Notably, emodin demonstrated consistent MBCs of 512 µg/ml. In comparison, ampicillin exhibited higher MIC values of 64, 128 µg/ml and 128 µg/ml against the same strains. Its bactericidal activity was also strain-dependent, with MBCs of 512, 1024 and 1024 µg/ml, respectively, reflecting a less uniform efficacy profile than emodin (see Table 1). According to the testing standard described in our previous study^31^and based on the microtiter plate assay, it was determined that all isolates of MRSA exhibited moderate biofilm production. It seemed that eradicating bacteria in the biofilm of MRSA were challenging, as the minimum biofilm inhibition concentrations (MBICs) and the minimum biofilm eradication concentration (MBBCs) were all more than 512 µg/ml for emodin alone and above 1024 µg/ml for Amp alone. Furthermore, the interaction between Amp and emodin was also determined among these *S. aureus* strains. And the results indicated a synergistic efficacy in the MRSA strain 18−9 and 19−13 (as shown in Table 2).

**Table 1:** Susceptibility of Amp or Emodin against the MRSA strains in suspension.

Strains	Amp (µg/ml)				Emodin (µg/ml)			
	MIC	MBC	MBIC	MBBC	MIC	MBC	MBIC	MBBC
MRSA 18 − 9	64	512	>1024	>1024	16	512	>512	>512
MRSA 19 − 10	128	1024	>1024	>1024	32	512	>512	>512
MRSA 19 − 13	128	1024	>1024	>1024	32	512	>512	>512

**Table 2:** Fractional inhibitory concentration index (FICI) values for combination between Amp and Emodin.

Strains	Amp (µg/ml)	Emodin (µg/ml)	FICI	Interpretation ^a^
MRSA 18 − 9	16	4	0.5	SYN
MRSA 19 − 10	4	16	0.53	IND
MRSA 19 − 13	4	8	0.28	SYN

^a^ For the FICI model, synergism (SYN) was determined as an FICI of ≤ 0.5, antagonism (ANT) as an FICI of >4.0 and indifference (IND) as an FICI of >0.5 to 4.0.

### Antibiofilm activity against *S. aureus* biofilm formation

**Fig. 1:**
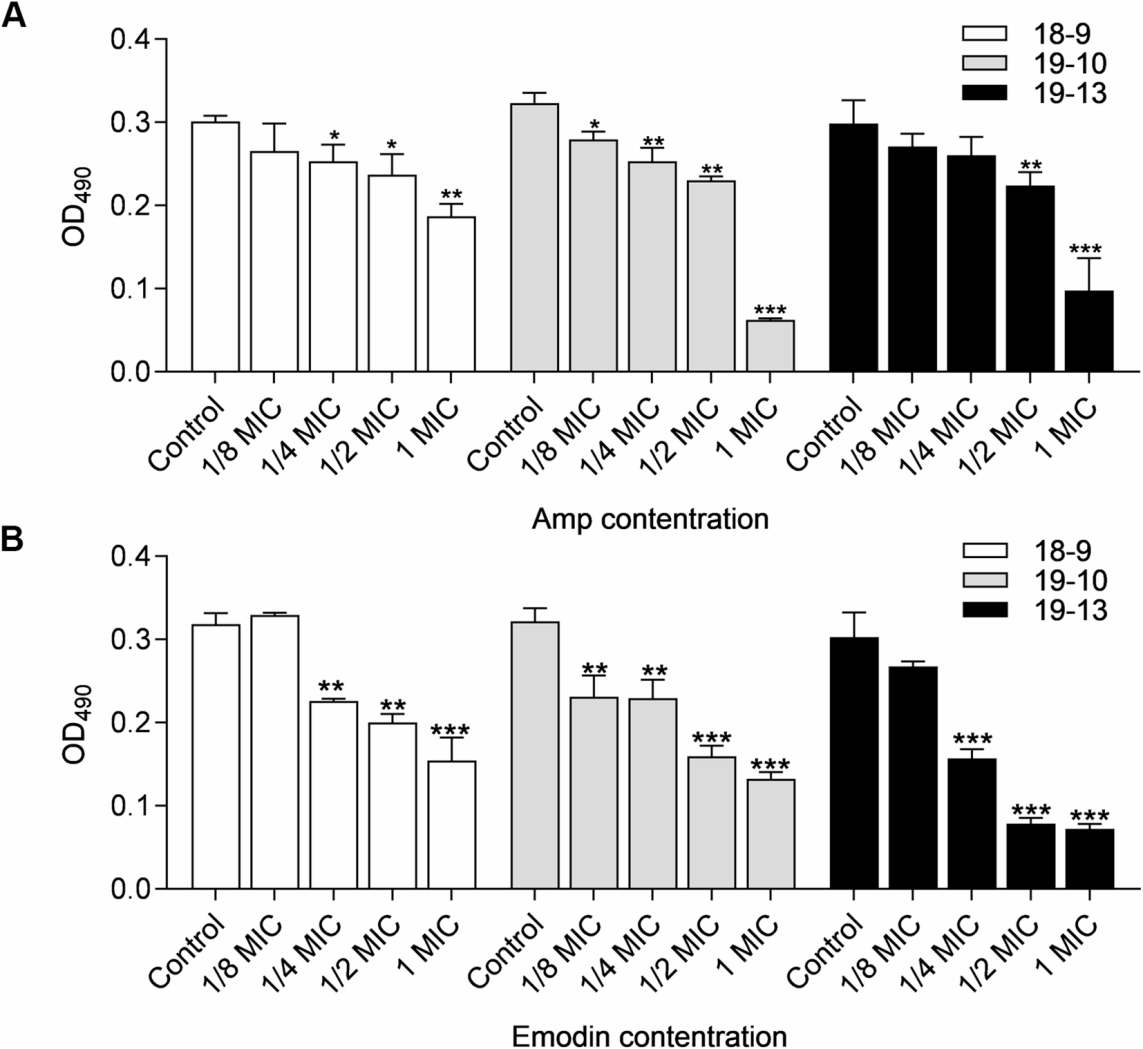
Effects of drugs on biofilm formation of MRSA strain. Biofilm formation by MRSA in presence of Amp (**A**) and emodin (**B**), in 96-well plates after 40–48 h, was assessed by crystal violet staining. Each bar indicates the mean values ± SE from at least three independent experiments. Control group means no Amp added in (**A**) and no emodin in (**B**) added. *p < 0.05, **p < 0.01, ***p < 0.001, compared with their respective control groups.

**Fig. 2:**
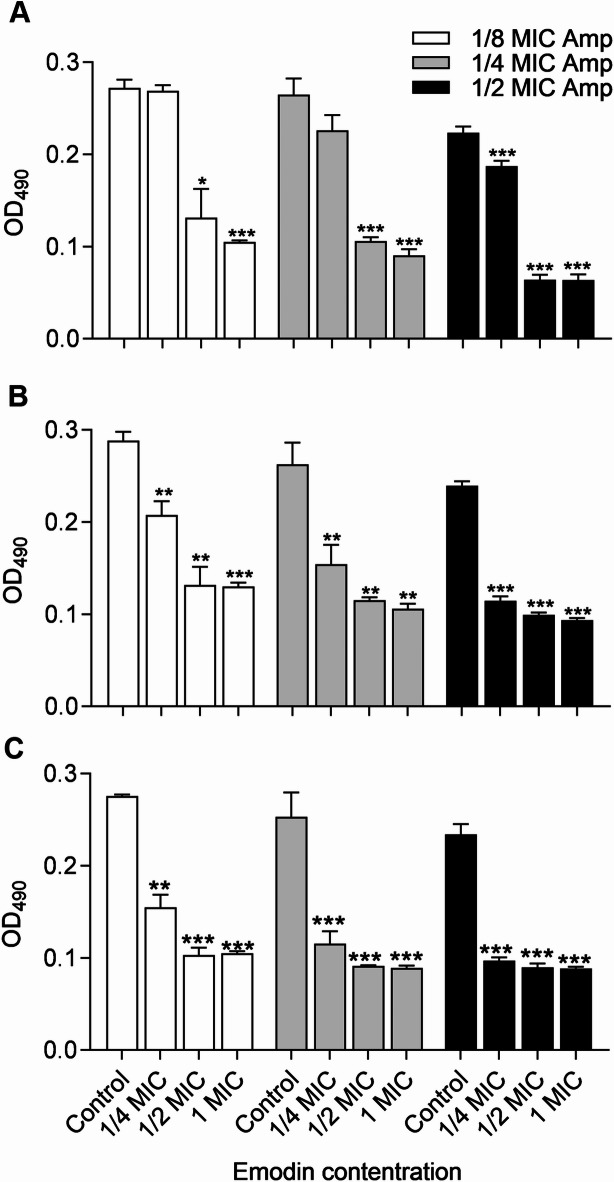
Combined effects of drugs on biofilm formation of MRSA strains. Biofilm formation by MRSA in presence of Amp and emodin, in 96-well plates after 40–48 h, was assessed by crystal violet staining. Each bar indicates the mean values ± SE from at least three independent experiments. Control group means no emodin added. *p < 0.05, **p < 0.01, ***p < 0.001, compared with their respective control groups.

MRSA biofilm formation was compared between strains cultured with and without drugs, specifically MICs and sub-MICs of Amp or emodin, for 48 h. As shown in Fig. 1B, emodin reduced the cell attachment in a dose-dependent manner across all strains. Moreover, at only 1/4 MIC, emodin markedly inhibited 29.03% (MRSA 18−9), 28.73% (MRSA 19−10) and 48.15% (MRSA 19−13) of biofilm formation, similar to the reduction observed with up to 1/2 MIC of Amp under the same conditions (Fig. 1A, B). Hence, this suggests that emodin may be more effective than Amp in disrupting *S. aureus* biofilm formation. To determine the combined efficacy, three major groups were exposed to 1/8 MIC, 1/4 MIC and 1/2 MIC of Amp alone. In addition, *S. aureus* in each group was treated with (1/4 to 1 MIC) or without emodin simultaneously. Various combinations yielded diverse consequences for these three MRSA strains. However, as depicted in Fig. 2, the addition of sub-MIC emodin in each group significantly enhanced the inhibition efficacy of biofilm formation, with more than 55% biofilm inhibition observed in the presence of 1/2 MIC emodin in every group.

### Combined efficacy in mature biofilms of *S. aureus*

**Fig. 3:**
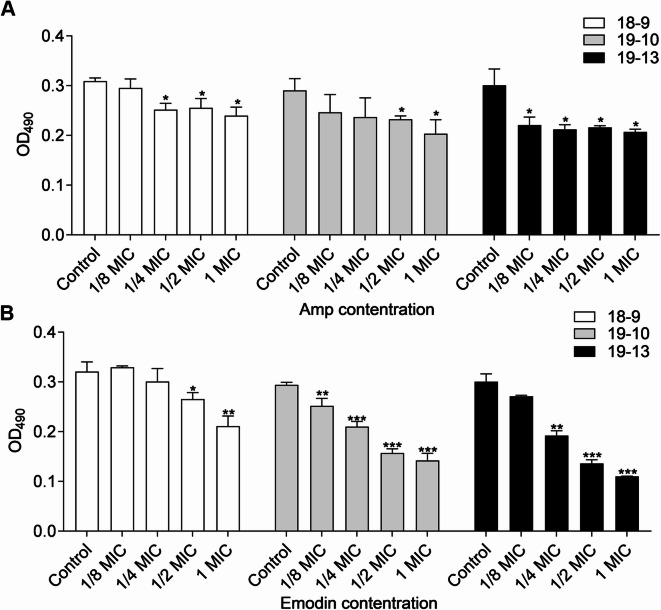
Effects of drugs on biofilm disruption of MRSA strain. Mature biofilm, incubated with fresh TSB + 0.5% glucose media containing Amp (**A**) and emodin (**B**) for another 40–48 h, was assessed by crystal violet staining. Each bar indicates the mean values ± SE from at least three independent experiments. Control group means no Amp added in (**A**) and no Emodin added in (**B**). *p < 0.05, **p < 0.01, ***p < 0.001, compared with their respective control groups.

**Fig. 4:**
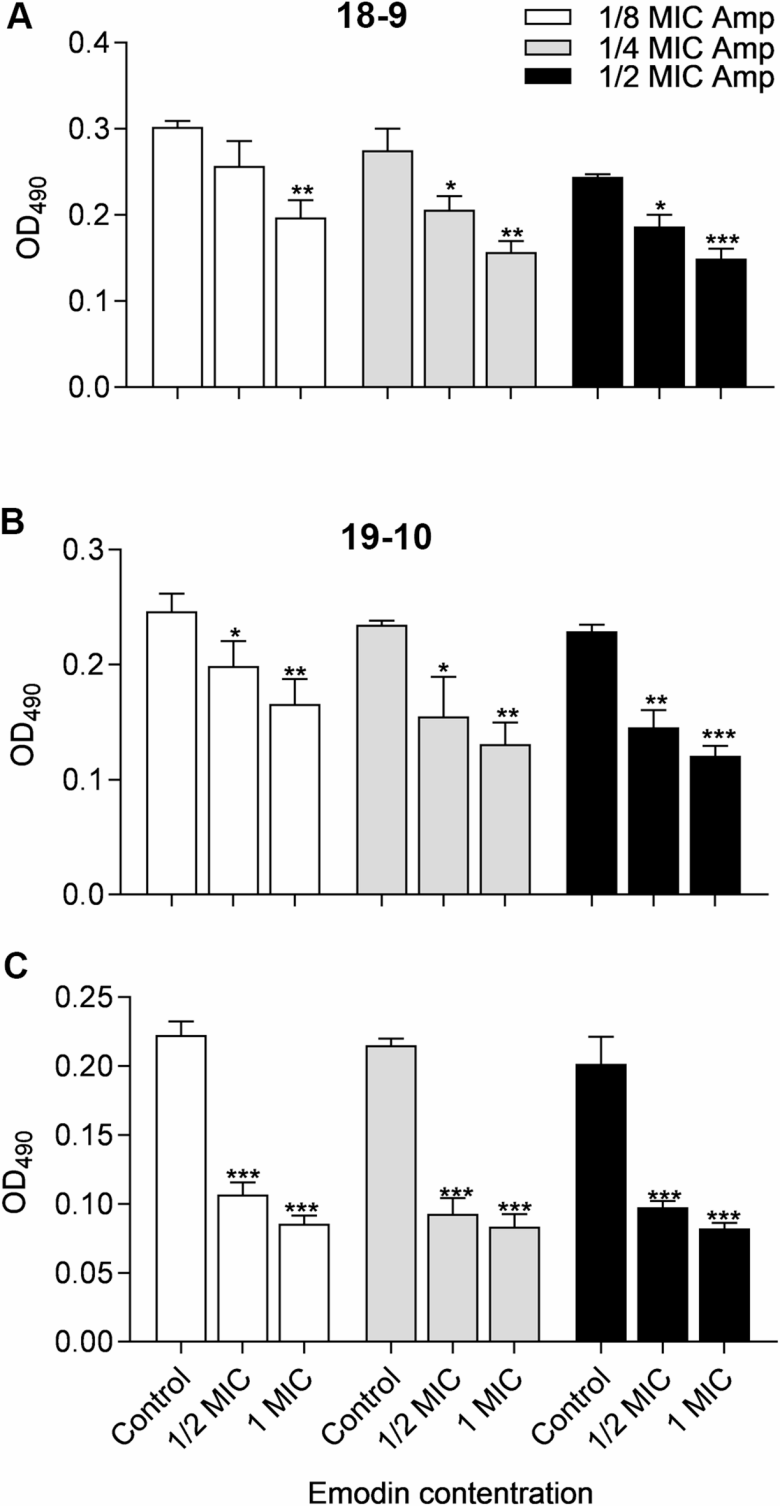
Combined effects of drugs on biofilm disruption of MRSA strains. Mature biofilm, incubated with fresh TSB + 0.5% glucose media containing Amp and emodin for another 40–48 h, was assessed by crystal violet staining. Each bar indicates the mean values ± SE from at least three independent experiments. Control group means no emodin added. *p < 0.05, **p < 0.01, ***p < 0.001, compared with their respective control groups.

In a second set of experiments, the disruption of the mature biofilms (48 h of growth in the absence of drugs) by the addition of Amp plus emodin at different concentrations for another 48 h was evaluated. Figure 3A illustrated that increasing the concentration of Amp had little impact on reducing the biofilm formation of the tested MRSA strains. In contrast, a dose-dependent decrease in biofilm levels was observed with increasing concentrations of emodin (Fig. 3B). The combined efficacy of Amp and emodin in destroying preformed biofilms also varied depending on the strain. However, at 1/2 MIC, compared to the controls, emodin exhibited a maximum of 50% (MRSA 19−13), 42% (MRSA 19−10), 24% (MRSA 18−9) inhibition of biofilm formation (Fig. 4).

### Observation of MRSA biofilm morphology by SEM

**Fig. 5:**
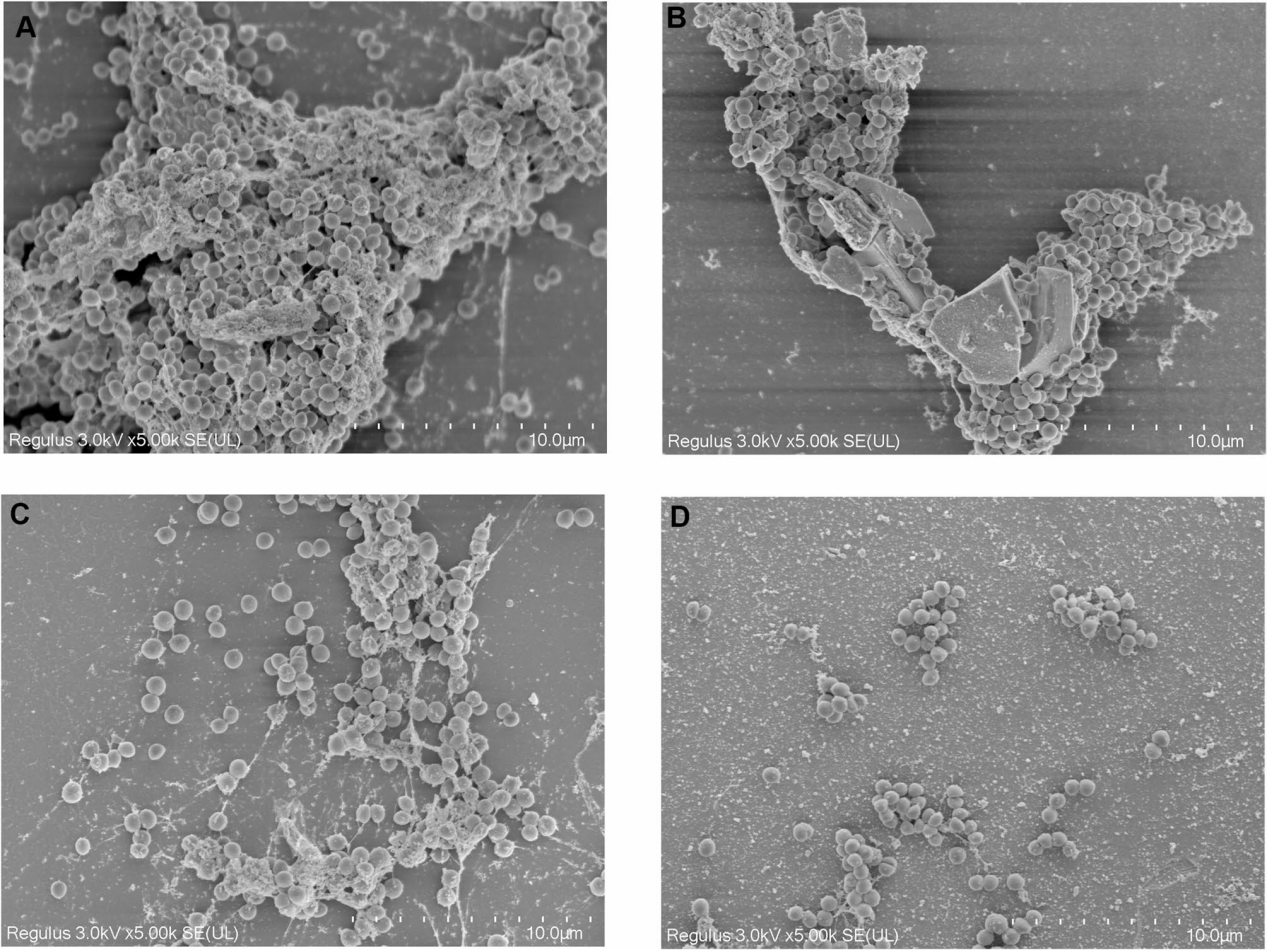
SEM analysis of MRSA 19−10 biofilm. SEM images of biofilm formed by MRSA 19−10 that had been incubated with 1/4 MIC of Amp together with 1/4 to 1 MIC concentrations of emodin for 40–48 h. Scale bars in the figures represented 10 μm.

Figure 5 displayed the morphology of the bacterial biofilm captured by SEM. In the control group, which received only 1/4 MIC of Amp, the bacteria were predominantly observed as dense cellular aggregates throughout the entire field of view. As the concentration of emodin was increased (ranging from 1/4 to 1 MIC) in the control group, the substances were largely, but not entirely, eliminated, aligning with the quantitative findings presented in Fig. 2.

### Gene expression analysis

**Fig. 6:**
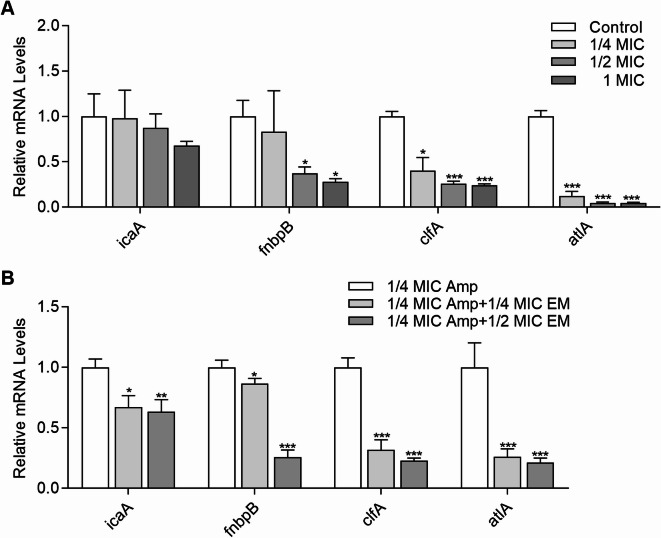
Expression of biofilm-related genes in MRSA 19−10 in response to emodin or in combination with Amp. The normalized fold expression changes in biofilm-related genes following exposure to emodin alone or in combination with Amp for 24 h was plotted against control biofilms without exposure to Emodin (**A**) or with only exposure to Amp (**B**) using *gyrB* as the reference gene. Each bar indicates the mean values ± SE from at least three independent experiments. *p < 0.05, **p < 0.01, ***p < 0.001, compared with their respective control groups.

To explore whether the influence manifested the transcriptional levels, the expression of the *ica* operon and important adhesion and eDNA-related genes were detected using RT-qPCR, and the results were shown in Fig. 6. Increasing the concentration of emodin did not result in a significant difference in the expression levels of *icaA* compared to the control blank (*p* > 0.05). However, *fnbpB*, *clfA* and *atlA* were downregulated in a dose-dependent manner (Fig. 6A). More importantly, the transcriptional levels of *fnbpB* and *clfA* were significantly decreased by 2.68- and 3.85-fold (*p* < 0.05) when exposed to 1/2 MIC emodin, respectively. Interestingly, the transcriptional levels of AtlA, the major *S. aureus* autolysin protein, were markedly inhibited by 8- to 21-fold after treatment with all subinhibitory concentrations of emodin (*p* < 0.001). Figure 6B illustrated the gene activity following exposure to a combination of Amp and emodin. Compared to the control group exposed solely to 1/4 MIC Amp, the expression levels of *icaA*, *fnbpB*, *clfA* and *atlA* were significantly decreased when both drugs were administered together. Similar to the results shown in Fig. 6A, the addition of 1/4 and 1/2 MIC emodin in the culture medium exhibited great significance in the transcript levels of *clfA* and *atlA* (*p* < 0.001).

### Analysis of slime production

**Fig. 7:**
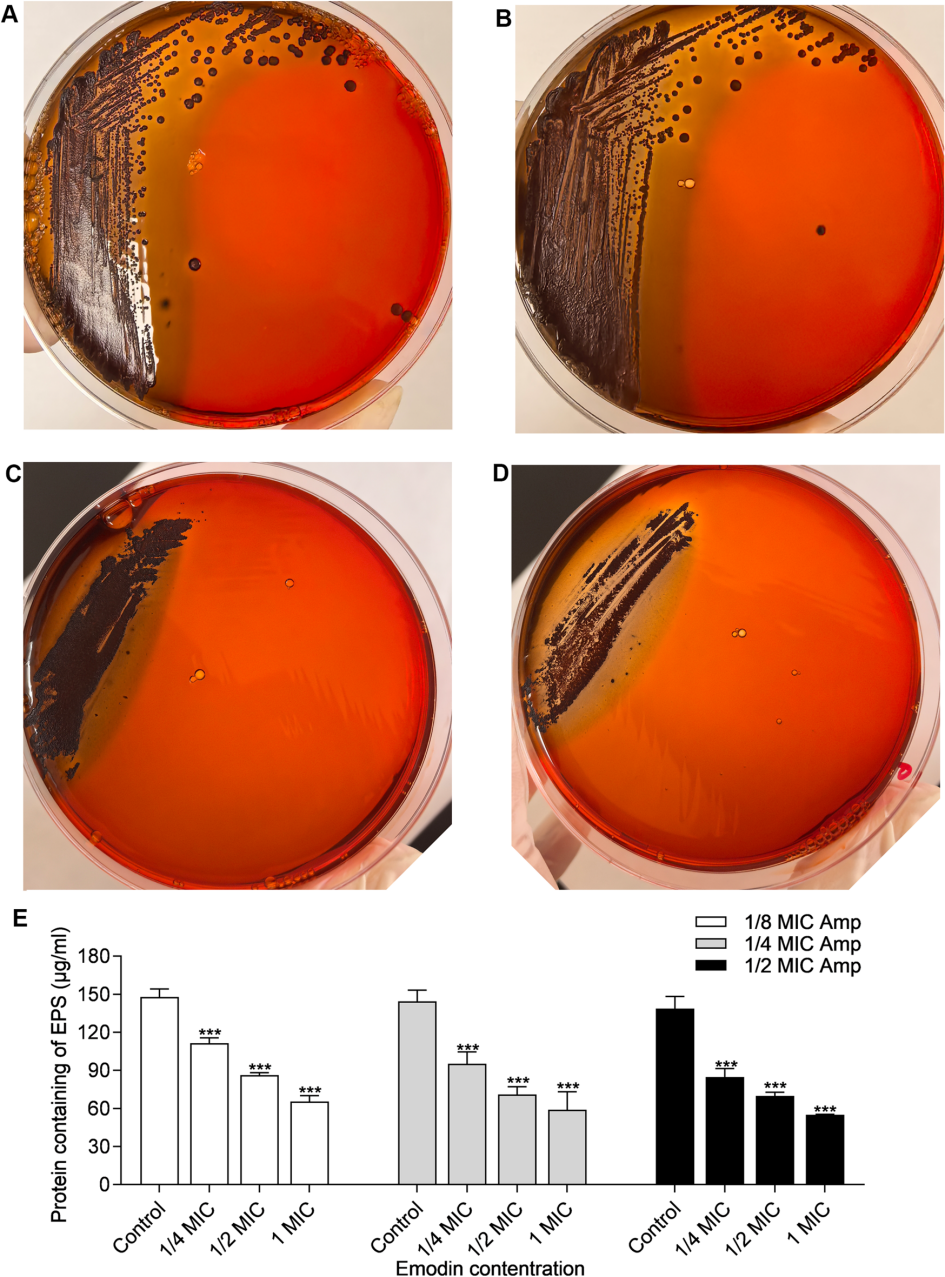
Effect of drugs on PIA formation and on extracellular proteins. (**A**) MRSA 19−10 was grown on Congo red medium and incubated with 1/4 MIC of Amp together with 1/4 to 1 MIC concentrations of emodin; (**B**) Analysis the effect of drugs on extracellular proteins by micro BCA method. Each bar indicates the mean values ± SE from at least three independent experiments. *p < 0.05, **p < 0.01, ***p < 0.001, compared with their respective control groups.

In *S. aureus*, the production of slime, which encompasses polysaccharide substrates, is intricately associated with the formation of biofilms. When Congo Red interacts with these polysaccharide substrates, it elicits a color transformation from red to black upon incubation. The findings revealed that the black pigmentation surrounding the colonies remained unaltered across varying concentrations of emodin (Supplementary Fig. 1) and when emodin was combined with 1/8 MIC Amp. However, a notably discernible reduction in coloration was observed when the colonies were treated with a combination of emodin and 1/4 MIC of Amp (Fig. 7A−D).

### Qualitative analysis of proteins in EPS

EPS is a complex mixture primarily composed of polysaccharides, proteins, lipids, and nucleic acids. These components interact to form a highly hydrated and dynamic matrix that provides structural support and functional properties to the biofilm. The adhesive properties of EPS enable bacteria to adhere to surfaces, forming the foundation for biofilm development. To further understand the role of proteins in EPS, Micro BCA Protein Assay was used to compare the content of proteins between the different treatment of the antimicrobial drugs. As shown in Fig. 7E, a notable decrease in the amount of protein was observed in MRSA cells treated with 1/4 to 1/2 MIC emodin, compared to cells treated only with Amp in each group (*p* < 0.001).

### Qualitative analysis of eDNA release and autolysis assay

**Fig. 8:**
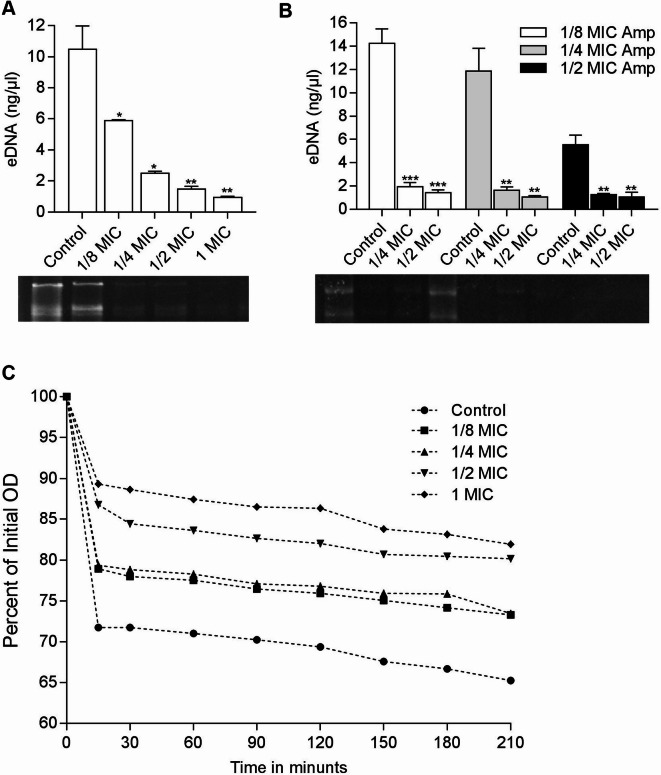
Effects of drugs on eDNA release and autolysis of MRSA strain 19−10. (**A**-**B**) The amount of eDNA in the cell-free supernatants from MRSA 19−10 biofilms treated with emodin alone or in combination with Amp was measured by spectrophotometry (upper panel) and agarose gel electrophoresis (down panel); **C**) Triton X-100 was used to stimulate autolysis in MRSA 19−10 cells grown in the absence or presence of various concentrations of emodin. The data were from a single representative experiment and were reproduced at least three times.

According to previous studies, eDNA, which is released through the autolysis of a small population of biofilm cells, constitutes a critical substrate in biofilms during the initial bacterial adhesion and surface aggregation. In this study, a notable decrease in the amount of eDNA release was observed in MRSA 19−10 cells treated with emodin at concentrations ranging from 1/4 to 1 MIC, compared to control cells (Fig. 8A). The amounts of eDNA in the cell-free supernatants from biofilms treated with the combination of antimicrobial agents were shown in Fig. 8B. Remarkably, a great reduction observed in eDNA release from biofilms was observed when treated with 1/4 to 1/2 MIC emodin, compared to cells treated only with Amp in each group (*p* < 0.001). To further assess the effect of emodin, MRSA 19−10 cells were treated with various concentrations of emodin, and the rate of autolysis induced by the nonionic detergent Triton X-100 was compared an untreated control culture (Fig. 8C). After 210 min, the OD_600_ values of the cells treated with 1/8 MIC, 1/4 MIC, 1/2 MIC and 1 MIC emodin were 73.30%, 73.46%, 80.16% and 81.94% of the initial value, respectively. In contrast, the control group displayed highly active autolysis, with an OD_600_ value of 65.27%.

## Discussion

The remarkable tolerance of bacteria in biofilms to antibiotics, host defense, nutritional deficiency and heat shock underscores the urgent need for the development of antibacterial agents or treatment options to address the chronic and recurrent infections that they cause. With advanced methodologies for separation and isolation procedures, an increasing number of natural products from plants have been considered candidates for biofilm-associated infections. In the current study, emodin alone or in combination with Amp was found to exhibit anti-MRSA biofilm activity through the downregulation of biofilm-related genes (*icaA*, *fnbpB*, *clfA* and *atlA*).

β-lactam antibiotics have traditionally been the preferred treatment for serious invasive infections caused by *S. aureus* until the emergence of MRSA, which has acquired a genetic element called *mecA*. *mecA* encodes an alternative penicillin-binding protein 2a (PBP2a) with diminished affinity for β-lactam antibiotics. However, MRSA susceptible to combinations of a β-lactam and compounds that disrupt the essential scaffold for PBP2a integrity^32^. Consequently, we have selected Amp as a therapeutic partner for emodin to combat MRSA biofilm activity. Our intention is to augment the anti-MRSA biofilm efficacy of the two antimicrobial agents by employing a combination therapy strategy.

A previous study had already investigated the effects of emodin on biofilm formation of *S. aureus* CMCC26003, a standard MSSA strain^30^. However, the antibiofilm activity of emodin alone or in combination with β-lactam antibiotics against MRSA is poorly understood. The MIC of emodin against *S. aureus* was reported to be 8 µg/ml and 7.8 µg/ml in previous studies^30^. In this work, the MIC against MRSA planktonic cells was 16–32 µg/ml (Table 1). This discrepancy in MIC values may be attributed to the varying bioactivity of emodin yielded by different manufacturers and distinct gene backgrounds. It is well known that MRSA is generally more resistant to antibiotics compared to MSSA^33^. Our results showed that emodin exhibited a concentration-dependent antibiofilm efficacy against all tested strains and showed a greater inhibition in the formation of biofilms at the same fold of MIC than that of Amp (Fig. 1A, B). As shown in Fig. 2, the combination of 1/4 MIC or 1/2 MIC Amp plus 1/2 MIC emodin significantly affected the biofilm formation of the tested strains. Importantly, the biofilms formed by MRSA strain 19−13 at all tested concentrations were significantly lower (*p* < 0.001) compared to the untreated controls. Therefore, we believed that emodin is capable of sensitizing Amp to MRSA biofilm formation in a dose-dependent manner.

In the clinic, biofilm-associated infections always occur without prevention^21^. Once biofilms have formed completely, they become more challenging to eradicate, requiring higher concentrations of antimicrobial agents due to the blocked diffusion^34^. Our findings, as indicated by crystal violet quantification, revealed that emodin was able to eradicate 28.55% of MRSA strain 19−10 and 34.75% of MRSA strain 19−13 mature biofilms only at 1/4-fold MIC. Beyond this concentration, the eradication effects became more pronounced, and statistical significance (*p* < 0.001) were observed. Additionally, an escalating inhibition ration against mature biofilms was observed with the increasing concentrations of both antimicrobial agents in MRSA strain. While the addition of 1/2 MIC emodin was enough to eradicate preformed biofilms, even a higher concentration of Amp (from 1/8 to 1/2 MIC) was required in MRSA strain 19−13.

To explore the molecular mechanism of emodin, the gene expression profiles of cells that were not treated and cells that were treated with emodin were compared using real-time qPCR analysis. *Staphylococcal* biofilms are surrounded by a self-produced extracellular matrix that consists of proteins, eDNA and polysaccharide intercellular adhesion (PIA). PIA synthesis is mediated by the *ica* operon. Therefore, downregulating *ica* gene expression could be an effective strategy to prevent *S. aureus* biofilm formation^30^. Nevertheless, it has been reported that MRSA strains can exhibit an *ica*-independent biofilm phenotype in vitro, while clinical MSSA isolates have been identified as PNAG-dependent biofilm phenotype^35^. Moreover, most MRSA biofilms consist of eDNA and adhesins, whereas MSSA strains typically form biofilms that contain polysaccharides in their matrices^9^. In our study, the CRA plate assay demonstrated that emodin had no discernible effect on slime production unless it was co-administered with 1/4 MIC Amp (Supplementary Fig. 1 and Fig. 7A-D). This finding was in accordance with the observation of a slight decrease in *ica* expression within the emodin-treated groups when Ampicillin was present at a sub-MIC level.

Microbial surface components recognizing adhesive matrix molecules (MSCRAMMs), such as fibronectin-binding proteins (Fnbps) and clumping factor A (ClfA), have been known to play an important role in the initial stage of biofilm formation^36,37^. Interestingly, Fnbps have been shown to compensate for the absence of PIA in facilitating biofilm formation in the *icaADBC*-independent biofilm phenotypes^36^. Moreover, this Fnbp-mediated biofilm is particularly common among highly virulent MRSA isolates, highlighting the significance of PIA-independent biofilm formation in MRSA strains. The micro-BCA method, derived from the established BCA method, exhibits enhanced sensitivity and is particularly well-suited for low-dose determinations. Our findings revealed a decreasing trend in the total protein content of EPS with an increasing concentration of emodin, within the sub-MIC range of Amp (as illustrated in the Fig. 7E). Consistent with previous studies^38–40^our results demonstrated that increased expression levels of *fnbpB* were associated with a reduction in biofilm formation. As shown in Fig. 6, the transcriptional levels of *clfA*, which shared 25% sequence identity with *fnbpB* in its A domain, were decreased by emodin in a dose-dependent manner.

The importance of eDNA within biofilms potentially hinges on its multifaceted roles: facilitating antibiotic resistance mechanisms, guiding nutrient localization during periods of starvation, and serving as a reservoir for the gene pool that enables horizontal gene transfer^41,42^. In the case of MRSA strains, eDNA release in biofilms is predominantly mediated by the autolysin protein AtlA, which is produced when a small population of biofilm cells undergo autolysis^43^. Initially described as a secreted enzyme responsible for maintaining cell wall metabolism during cell division and growth, AtlA’s participation in biofilm development has been demonstrated in various biofilm models^43^. The role of AtlA-mediated lysis in biofilm development was revealed in some biofilm models^44^. In our study, we observed a significant 7.1- and 4.2-fold decrease in the expression of the *atlA* gene when exposing the cells to a concentration of only 1/4 MIC emodin (Fig. 6), both in the absence or presence of 1/2 MIC Amp, aligning with the observed changes in eDNA levels (Fig. 8).

Pharmacological investigations have underscored the therapeutic promise of emodin in traditional medicine for managing conditions such as inflammation and cancer. Concurrently, accumulating evidence of its organ-specific toxicity, particularly hepatotoxicity and nephrotoxicity, had prompted heightened safety concerns in clinical applications^45,46^. Concomitant administration of two or more pharmacological agents, synergistically augment treatment efficacy while attenuating systemic toxicities. This combinatorial strategy confers multifaceted benefits, including enhanced therapeutic index, reduced risk of drug resistance development, and improved patient compliance through tailored dosing paradigms. Our study, which investigated the combinatorial anti-biofilm activity of emodin and ampicillin against MRSA through a synergistic administration paradigm, constituted an exploratory approach to concurrent drug delivery strategies. On the other hand, nanotechnology-based drug delivery platforms have emerged as revolutionizing pharmaceutics by enhancing drug solubility, improving biocompatibility, extending circulation duration, and reducing toxicity through targeted design. A recent study demonstrated that the in situ delivery of emodin via Pluronic F-127 not only enhances its aqueous solubility but also enables targeted, prolonged release at the targeted site^47^. Subsequently, we will aim to elucidate the pronounced anti-biofilm efficacy of emodin through a targeted nanoparticle delivery system. On the other hand, the combination of other antibiotics, such as rifampicin and tigecycline, with emodin will be explored to investigate their synergistic effects against MRSA biofilms.

In summary, the present work demonstrated the potential of emodin, an anthraquinone derived from *R. palmatum*, to prevent biofilm formation and disrupt the mature biofilm of MRSA strains alone or in combination with Amp. This inhibitory effect was observed through the downregulation of *fnbpB*, *clfA* and *atlA* genes. Unfortunately, to date, no reliable animal model of biofilm infection has been established, which has precluded us from further validating the synergistic anti-MRSA biofilm infection effect of emodin and ampicillin in vivo. This limitation will be a critical focus of our future research endeavors.

## Methods

### Bacterial strains and reagents

In this study, all *S. aureus* isolates were collected from patients at the local hospital in Hangzhou city. The detailed information pertaining to these strains was presented in Supplementary Table 1. The antibiotic resistance profiles of these isolates were tested against 16 drugs, as recommended by the Clinical and Laboratory Standards Institute (CLSI) criteria. Three MRSA strains, exhibited resistance to oxacillin with a MIC of 8 µg/ml and were subsequently identified as MRSA through detection of the specific *mecA* gene. Bacterial stocks of each strain were maintained at −80 °C in tryptic soy broth (TSB) containing 20% glycerol (v/v). To initiate experiments, all the strains were thawed and subcultured in tryptic soy agar (TSA) for 18–24 h.

Emodin and ampicillin (Amp), both procured from Sangon Biotech (Shanghai, China), were prepared as stock solutions at a concentration of 64 mg/ml. Specifically, emodin was dissolved in DMSO (Sigma-Aldrich, St. Louis, MO, USA), while Amp was reconstituted in ultrapure water.

### Antimicrobial activity of *S. aureus* in suspension

The MICs of emodin and Amp against a standard strain and three MRSA isolates were assessed using the broth microdilution method. MRSA cultures were grown in Mueller-Hinton broth (MHB) at 37 °C with shaking for 4–8 h until the exponential growth phase (0.5 McFarland) was achieved. Serial two-fold dilutions of emodin (2–64 µg/ml) and Amp (2–256 µg/ml) were prepared in MHB in a 96-well plate (100 µl/well). A diluted bacterial suspension was added to each well to attain a final concentration of 1 × 10⁵ CFU/ml. Negative controls consisted of wells with only MHB, while positive controls had MHB with inoculated bacteria but no emodin. The plates were incubated at 37 °C for 24 h. The MIC was expressed as the lowest concentration that showed no visible growth in the medium. After establishing the MIC values, samples from wells showing no visible growth were subcultured onto Mueller-Hinton agar (MHA) plates. These plates were then incubated at 37 °C for 24 h. The MBC was defined as the lowest concentration that showed no microbial growth on agar. Each isolate in each drug was tested in triplicate.

### Interactions between Amp and emodin against *S. aureus* in suspension

A checkerboard microdilution method was employed to examine the combined efficacy of Amp and emodin against MRSA. Serial twofold dilutions were prepared in Mueller Hinton (MH) broth, covering a range from 1/32- to 4-fold MIC for Amp, and from 1/64- to 2-fold MIC for emodin. The 96-well plates were incubated at 37 °C for 18–24 h. To evaluate the effect of the combination, the fractional inhibitory concentration index (FICI) was computed using the following formula^48^:

FICI_A_= MIC of A in combination/MIC of A alone.

FICI = FICI_Amp_ + FICI_Emodin_.

FICI ≤ 0.5, synergy; 0.5 < FICI ≤ 4.0, indifference; FICI > 4.0, antagonism.

### Establishment of biofilms

Biofilm formation was measured as previously described^31^. Briefly, individual clones were cultivated in TSB and incubated in an orbital shaker (180 rpm) at 37 °C for 6 h. 1% of the *S. aureus* culture was used for all assays. The bacterial cultures were adjusted to a turbidity of 0.5 McFarland scale using phosphate buffered saline (PBS). Subsequently, the cultures were diluted 1:100 into TSB supplemented with 0.5% glucose and added to each well of a sterile 96-well flat-bottom microtiter plate (Corning Incorporated, Corning, New York, USA), which was incubated at 37 °C for 40–48 h under static conditions. Following incubation, the planktonic cells were removed by washing, and the remaining adherent bacterial cells in each well were stained with 100 µl of 0.1% crystal violet solution (Sangon Biotech, Shanghai, China). To dissolve the plates, 100 µl of 33% glacial acetic acid (v/v) was added to per well, and the absorption was subsequently measured at 490 nm using an iMark microplate absorbance reader (Bio-Rad Laboratories, Hercules, California, USA).

### Antimicrobial activity of *S. aureus* in biofilms

Cultivated biofilms were gently washed with PBS to remove planktonic cells, and then incubated for an additional 24 h at 37 °C in the presence of antimicrobial agents. Subsequently, the antimicrobial drugs were then removed and the biofilms were washed. PBS-treated biofilms were served as a positive control. The biofilms on the bottom were scrapped and washed with 250 µl PBS, while the contents of wells (10 µl) were mixed with warm MH broth and incubated for 24 h at 37 °C. The MBIC was defined as the lowest concentration that inhibited visible growth of the bacteria in the medium. The samples that exhibited no growth on agar at the lowest concentration were recorded as the MBBC^49^.

### Biofilm disruption

To determine the impacts on mature biofilms, *S. aureus* was allowed to form biofilms on 96-well plates in the absence of the aforementioned drugs. Subsequently, the nonadherent cells were removed, and fresh TSB + 0.5% glucose media, along with various concentrations of agents, were added to each well independently or in combination for an additional 40–48 h at 37 °C. The amounts within biofilms were quantified using crystal violet staining as described above.

### Scanning electron microscopy assay

MRSA cells (1.0 × 10^6^ CFU/ml) were cultivated in fresh TSB + 0.5% glucose media, housed in 12-well plates with glasses coverslips. Various sub-MIC concentrations of emodin, along with 1/4 MIC of Amp, were introduced into the wells and incubated at 37 °C. Following an incubation period ranging from 40 to 48 h, the supernatant was discarded, and the biofilms on the coverslips were washed gently with PBS. Subsequently, 2.5% glutaraldehyde was administered to fix the biofilms at 4 °C overnight. After washing with PBS, the samples were dehydrated through a series of ethanol concentrations (30%, 50%, 70%, 80%, 95%, and 100%), with each step lasting 10 min each. The coverslips were dried with CO_2_ in a critical-point drier (Emitech K850, Ashford, England), coated with gold (Hitachi E1045, Tokyo, Japan). The samples were taken for observation with a scanning electron microscope (Hitachi Regulus8100, Tokyo, Japan) and images were acquired at an accelerating voltage of 5 kV.

### RNA isolation and real-time PCR analysis

Bacterial cells scraped from MRSA biofilms that were treated with antimicrobial agents alone or in combination, were suspended in 25 µg/ml lysostaphin (Sigma-Aldrich, St. Louis, Missouri, USA) and incubated at 37 °C for 3 h. Then, RNA extraction was performed using the RNeasy Mini Kit (Qiagen, Dusseldorf, Germany) according to the manufacturer’s instructions. The concentration of RNA was quantified using a NanoDrop spectrophotometer. cDNA was obtained by transcription of 150 ng of the total RNA using PrimeScript™ RT Master Mix (Takara, Tokyo, Japan). Real-time PCR analysis was performed on a thermal cycler (Roche Group, Basel, Switzerland) for the genes *icaA*, *fnbpB*, *clfA* and *atlA* using PCR mix (Takara, Tokyo, Japan) at a predefined ratio. Cycle threshold (Ct) values of all the tested genes were normalized using the Ct value of the housekeeping gene *gyrB* (*gyrase B*). Finally, the expression levels were quantified by the 2^(–ΔΔCt)^ method^39^. Primer sequences of the genes used in this study are given in Supplementary Table 2.

### Slime production assay

Slime production assays were conducted using Congo Red agar (CRA), as previously described^50^.Congo red agar (CRA) plates consisted of brain-heart infusion (37 g/L), sucrose (36 g/L), and agar (15 g/L). After autoclaving the medium, the separated Congo Red dye (0.8 g/L), together with control and the antimicrobial drugs were added to the agar medium when the temperature was cooled to 55 °C. The mixture was then poured into plates and allowed to solidify for use. Overnight cultures of MRSA cells (10 µl) were dropped on CRA plates and incubated for 24 h at 37 °C before imaging. Three independent experiments were conducted.

### EPS extraction and analysis

The EPS was extracted by using a modified method as described^51^. Briefly, the biofilms of *S. aureus* were grown in 96-well microtiter plates (Corning, Costar, USA) treated with different concentrations of emodin or a combination with ampicillin for 24 h. The medium was discarded, and the biofilms was washed with PBS before dissolved in PBS solution to prepare EPS. Protein in EPS extractions was determined quantitatively using the Micro BCA Protein Assay Kit (Sangon Biotech, Shanghai, China) according to the manufacturer.

### Extraction of eDNA

eDNA was extracted from biofilms and quantified using a modified version of the method described in a previous study. Briefly, the biofilms of *S. aureus* were grown on 6-well plates and treated with either emodin alone or a combination with ampicillin for 24 h. After chilling at 4 °C for 1 h, 10 µl 0.5 M EDTA was added. The medium was discarded, and the unwashed biofilms were scraped and resuspended in Tris-EDTA (TE) buffer (10 mM Tris, 1 mM EDTA). The suspension was vigorously vortexed for 1 h. After centrifugation (14000 rpm for 10 min), the supernatants were transferred to new tubes, and the remaining bacteria were removed via a 0.22 μm filter (Millipore Corporation, Billerica, Massachusetts, USA). The filtered supernatants were subjected to 1.5% (w/v) agarose gel electrophoresis and quantified by mixing 10 µl of supernatant with fluorescent dyes from Qubit (Invitrogen Life Technologies, Carlsbad, California, USA) for quantifying the DNA. The fluorescence of the DNA-dye interaction was measured using a Qubit 2.0 Fluorometer according to the manufacturer’s instructions.

### Triton X-100-induced autolysis assays

Concentrations of 1/8 MIC, 1/4 MIC, 1/2 MIC or 1 MIC emodin were added to the cultures when the MRSA strain reached an optical density at 600 nm (OD_600_) of 0.3. The cultures were then incubated with shaking at 37 °C until the OD_600_ reached 0.7 in control cultures. The cells, including those treated with emodin, were harvested by centrifugation and washed once with cold distilled PBS. The resulting cell pellet was resuspended in 0.05 M Tris–HCl (pH 7.0) containing 0.05% (v/v) Triton X-100. Afterwards, the cell suspension was then incubated at 37 °C with shaking, and the OD_600_ was determined at various time intervals. 

### Statistical analysis

All experiments were carried out in triplicate, and values are presented as the mean ± standard deviation (SD). Statistical analysis was performed using Student’s t-test (*p < 0.05, **p < 0.01, ***p < 0.001).

## Supplementary Information

Below is the link to the electronic supplementary material.

Supplementary material 1 (JPG 733.3 kb)

Supplementary material 2 (TIF 475.5 kb)

Supplementary material 3 (DOCX 24.0 kb)

## Data Availability

The raw datasets generated and/or analyzed during the current study are available from the corresponding author upon reasonable request. For any inquiries or requests regarding data access, please contact chenqi_hz@aliyun.com.
