# Supplementary figures and images for: Antibiofilm efficacy of emodin alone or combined with ampicillin against methicillin-resistant Staphylococcus aureus
Source: Sci Rep. 2025 Jul 1;15:21904. doi: 10.1038/s41598-025-06800-5 (PMC12218329; doi:10.1038/s41598-025-06800-5)

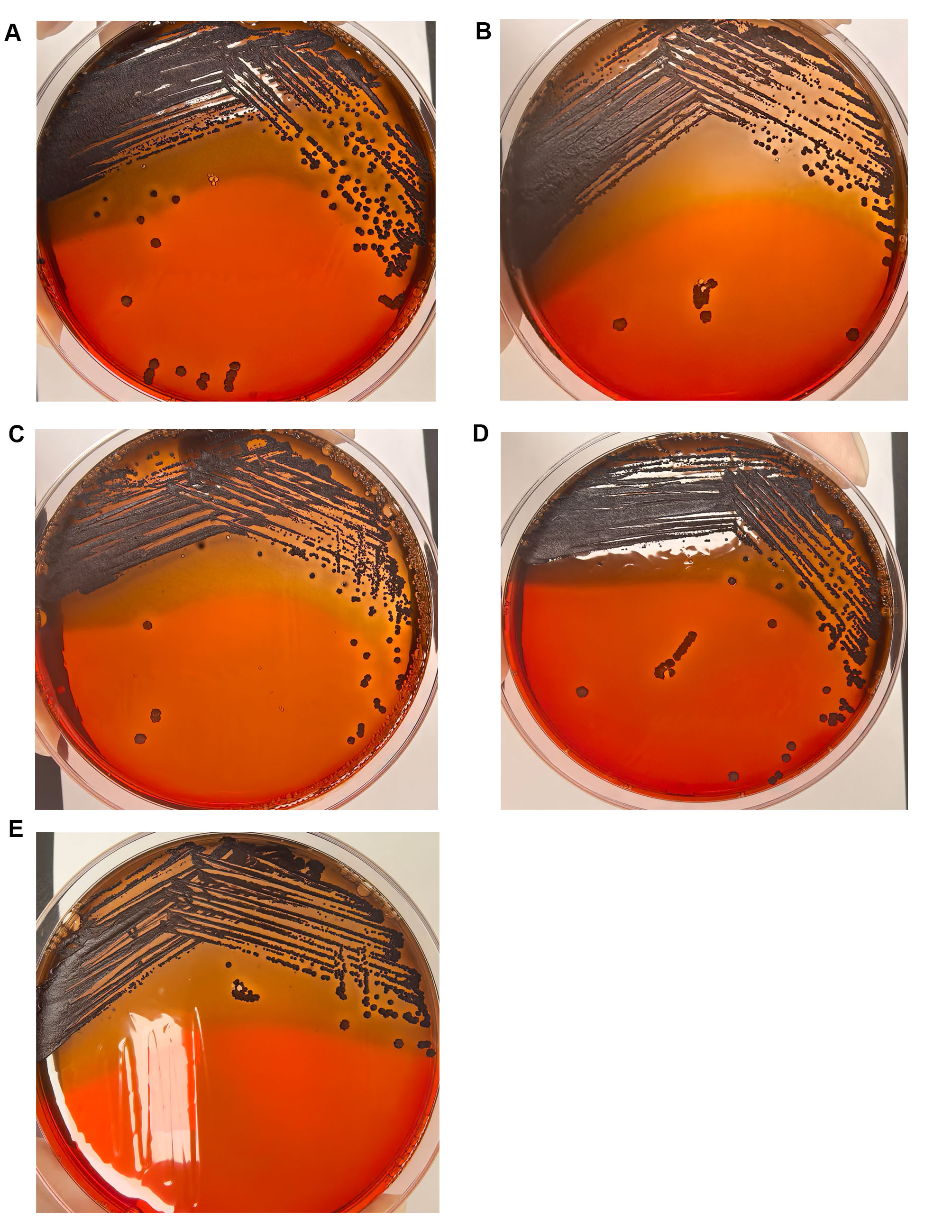

Supplement: Supplementary file 1 — Supplementary material 1 (JPG 733.3 kb) [file 41598_2025_6800_MOESM1_ESM.jpg]

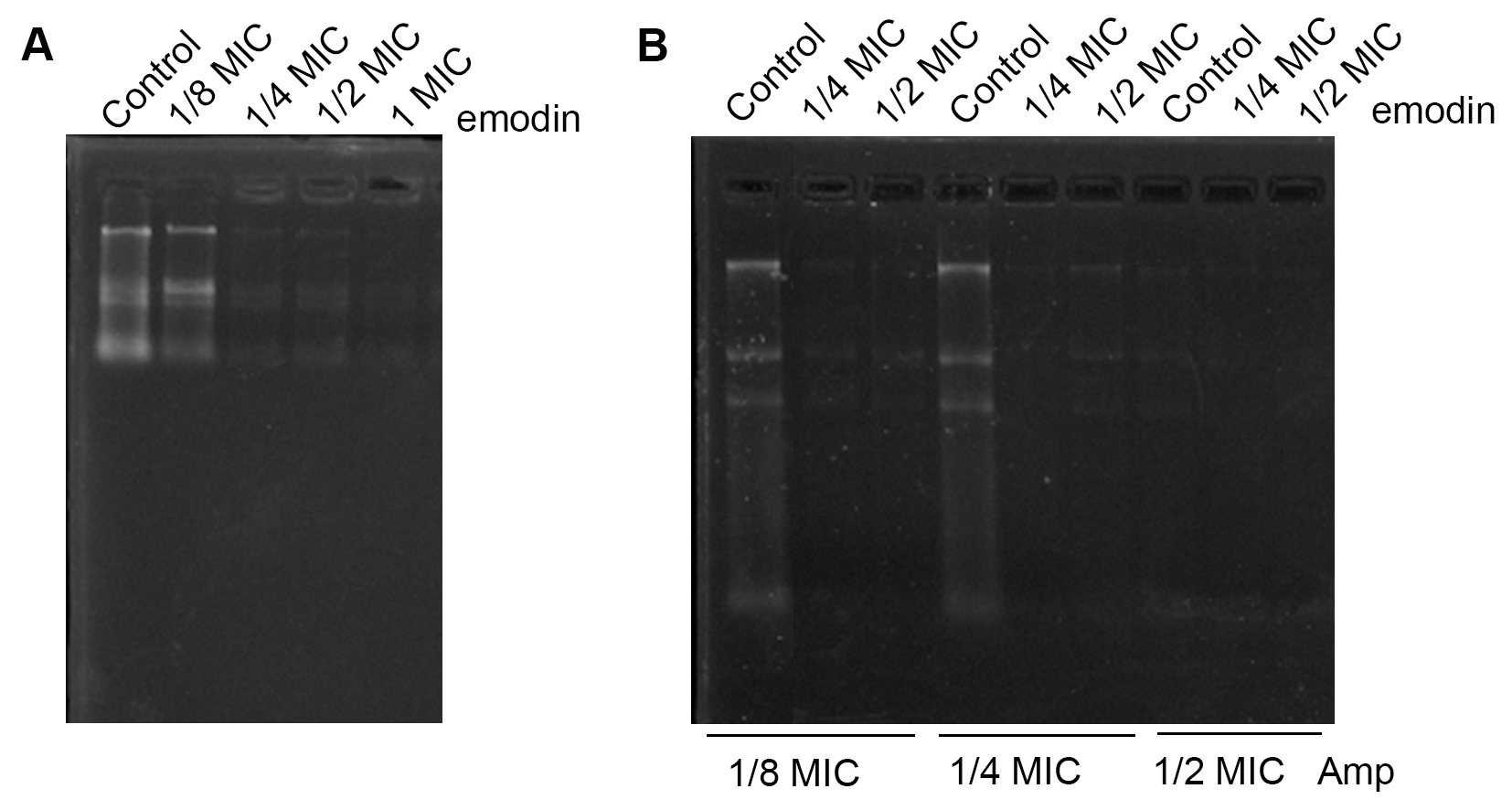

Supplement: Supplementary file 2 — Supplementary material 2 (TIF 475.5 kb) [file 41598_2025_6800_MOESM2_ESM.tif]
