# Supplementary material for: Antibiofilm efficacy of emodin alone or combined with ampicillin against methicillin-resistant Staphylococcus aureus
Source: Sci Rep. 2025 Jul 1;15:21904. doi: 10.1038/s41598-025-06800-5 (PMC12218329; doi:10.1038/s41598-025-06800-5)
Supplement: Supplementary file 3 — Supplementary material 3 (DOCX 24.0 kb) [file 41598_2025_6800_MOESM3_ESM.docx]

**Supplementary Table 1. Characteristics of patients and MRSA isolates**

| Strains | A, G | Admission diagnosis | Location of MRSA |
| --- | --- | --- | --- |
| MRSA 18-9 | 86, M | Respiratory failure | Blood |
| MRSA 19-10 | 75, M | Pneumonia | Sputum |
| MRSA 19-13 | 43, M | Cerebral hemorrhage | Sputum |

**Supplementary Table 2. List of primers used for qPCR analysis.**

| Gene | Nucleotide sequence (5’→3’) | Reference |
| --- | --- | --- |
| *gyrB* | GGTGCTGGGCAAATACAAGT | (Schwartz*, et al.*, 2016) |
|  | TCCCACACTAAATGGTGCAA |  |
| *icaA* | GTCAGACACTTGCTGGCGCA | (Chen*, et al.*, 2020) |
|  | GAGCCCATCTCACGCGTTGC |  |
| *fnbpB* | AAGAAGCACCGAAAACTGTG | (Jo and Ahn, 2016) |
|  | TCTCTGCAACTGCTGTAACG |  |
| *clfA* | ATTGGCGTGGCTTCAGTGCT | (Jo and Ahn, 2016) |
|  | CGTTTCTTCCGTAGTTGCATTTG |  |
| *altA* | TGTCGAAGTATTTGCCGACTTCGC | (Jo and Ahn, 2016) |
|  | TGGAATCCTGCACATCCAGGAAC |  |

**Supplementary Figure 1. Effect of emodin on PIA formation and on extracellular proteins.** A-E) MRSA 19-10 was grown on Congo red medium and incubated with 1/8 to 1 MIC concentrations of emodin.

**Supplementary Figure 2. Effects of drugs on eDNA release and autolysis of MRSA strain 19-10. A-B)** The amount of eDNA in the cell-free supernatants from MRSA 19-10 biofilms treated with emodin alone or in combination with Amp was measured by agarose gel electrophoresis.

**References**

Chen, Q., Xie, S., Lou, X., Cheng, S., Liu, X., Zheng, W.*, et al.* (2020) Biofilm formation and prevalence of adhesion genes among Staphylococcus aureus isolates from different food sources, *Microbiologyopen* **9**: e00946.

Jo, A., and Ahn, J. (2016) Phenotypic and genotypic characterisation of multiple antibiotic-resistant Staphylococcus aureus exposed to subinhibitory levels of oxacillin and levofloxacin, *BMC Microbiol* **16**: 170.

Schwartz, K., Ganesan, M., Payne, D.E., Solomon, M.J., and Boles, B.R. (2016) Extracellular DNA facilitates the formation of functional amyloids in Staphylococcus aureus biofilms, *Mol Microbiol* **99**: 123-134.
